# Rapid Design Optimization and Calibration of Microwave Sensors Based on Equivalent Complementary Resonators for High Sensitivity and Low Fabrication Tolerance

**DOI:** 10.3390/s23021044

**Published:** 2023-01-16

**Authors:** Tanveerul Haq, Slawomir Koziel

**Affiliations:** 1 Engineering Optimization and Modeling Center, Reykjavik University, 102 Reykjavik, Iceland; 2 Faculty of Electronics, Telecommunications and Informatics, Gdansk University of Technology, 80-233 Gdansk, Poland

**Keywords:** complementary resonators, design optimization, dielectric substrate, inverse modeling, microwave sensor, oils, permittivity

## Abstract

This paper presents the design, optimization, and calibration of multivariable resonators for microwave dielectric sensors. An optimization technique for the circular complementary split ring resonator (CC-SRR) and square complementary split ring resonator (SC-SRR) is presented to achieve the required transmission response in a precise manner. The optimized resonators are manufactured using a standard photolithographic technique and measured for fabrication tolerance. The fabricated sensor is presented for the high-resolution characterization of dielectric substrates and oil samples. A three-dimensional dielectric container is attached to the sensor and acts as a pool for the sample under test (SUT). In the presented technique, the dielectric substrates and oil samples can interact directly with the electromagnetic (EM) field emitted from the resonator. For the sake of sensor calibration, a relation between the relative permittivity of the dielectric samples and the resonant frequency of the sensor is established in the form of an inverse regression model. Comparisons with state-of-the-art sensors indicate the superiority of the presented design in terms of oil characterization reliability. The significant technical contributions of this work include the employment of the rigorous optimization of geometry parameters of the sensor, leading to its superior performance, and the development and application of the inverse-model-based calibration procedure.

## 1. Introduction

Metamaterials (MTMs) play a vital role in the development of microwave sensors. MTMs are artificially engineered electromagnetic materials in which metallic elements are arranged periodically to achieve extraordinary properties unavailable in conventional materials [1]. MTMs are classified into two categories: non-resonant and resonant. The non-resonant MTM technique, which is based on the transmission lines, was initially proposed by Iyer et al. [2], A. Oliner [3], and C. Caloz et al. [4] to achieve a negative refractive index medium and artificial left-handed materials. The resonant MTM technique is based on sub-wavelength resonant components such as split ring resonators (SRR) [5] and complementary split ring resonators (CSRR) [6]. The SRR was proposed by John Pendry et al. [7] as a resonant magnetic particle capable of achieving effective negative permeability. The CSRR is a dual counterpart of SRR and was proposed by F. Falcone et al. [8] as an effective negative permittivity resonator in 2004. In the following year, J.D. Baena et al. [9] introduced the equivalent circuit for the CSRR, which was based on lumped elements, that has since gained widespread acceptance. It has been demonstrated that CSRR can be excited by external electric and magnetic fields to exhibit a cross-polarization effect. This concept has been utilized to design a metasheet for transmission polarization [10], miniature antennas [11], waveguide passband filters [12], and microstrip stopband filters [13]. Later on, these filtering characteristics were used for microwave sensing applications [14].

Recently, CSRR-based microwave sensors have begun to attract renewed interest in the real-time characterization of microfluidic and solid materials [15,16,17,18,19,20,21,22,23,24]. This analysis is based on the alteration of the transmission response of the CSRR, more specifically, changing the operating parameters (resonant frequency, notch depth, and quality factor) due to its interaction with the sample under test (SUT). In [15], a CSRR was used to characterize high-energy materials with a resonant frequency (*f*_r_) of 4.48 GHz and a minimum frequency shift (Δ*f*_min_) of 20 MHz. The CSRR was applied in [16] to analyze a water-ethanol mixture with a *f*_r_ of 2.35 GHz and a Δ*f*_min_ of 50 MHz. In another study [17], a quasi-static CSRR was used for the nondestructive thickness measurement of Teflon films, with a *f*_r_ of 2.3 GHz and a Δ*f*_min_ of 151 MHz. In [18], an ethanol chemical sensor was proposed that employs a CSRR-loaded patch with a *f*_r_ of 4.16 GHz and a Δ*f*_min_ of 300 MHz. In another study [19], a square-shaped CSRR was utilized to measure the thickness of multilayer dielectric substrates with a *f*_r_ of 3.83 GHz and a Δ*f*_min_ of 348 MHz. In [20], a CSRR connected to a microstrip line was employed for microfluidic dielectric characterization with a *f*_r_ of 2 GHz and a Δ*f*_min_ of 400 MHz. In another study [21], a dual-band sensor was designed using a single compound CSRR to evaluate the thickness of dielectric substrates with an average error of 6.26 percent. In [22], two CSRRs were used for the noninvasive measurement of glucose levels with a concentration of 0 to 400 mg/dL, featuring a sensitivity of 0.03 dB per mg/mL. In another study [23], a multiple-squares CSRR-loaded flared patch was introduced to evaluate the oils, with a *f*_r_ of 8.49 GHz and a Δ*f*_min_ of 885 MHz. In [24], a square CSRR-loaded microstrip patch was presented to evaluate edible oils, with a *f*_r_ of 9.80 GHz and a Δ*f*_min_ of 1133 MHz. The aforementioned microwave sensors were designed to operate at frequencies lower than 10 GHz to avoid fabrication and measurement challenges. Notwithstanding, the sensor’s resonant frequency, sensitivity, and range of applicability are highly dependent on the appropriate sizing of the circuit dimensions. Typically, the sensitivity of microwave sensors based on CSRR is determined by the resonant frequency, the material properties of the SUT, and the interaction of the SUT with the CSRR’s electromagnetic field. When designing a high-sensitivity sensor, these three parameters are to be considered while staying within the fabrication and measurement limits. Interactive methods such as parameter sweeping are used in practice to realize the tuning of parameters [25,26]. In [25], the parametric sweep method was used to study the effects of resonator dimensions on the resonance frequency and notch depth of a filter by varying one parameter while holding the others constant. In [26], four geometric parameters were utilized to tune the resonant frequency of the CSRR, utilizing the parametric sweep technique. This method is computationally inefficient since it requires a full electromagnetic (EM) wave simulation for each geometrical parameter combination, which is a time-consuming process. This method involves the designer’s direct participation to produce the desired outcomes, resulting in suboptimal results. Therefore, an optimization approach is required to simultaneously manage numerous geometric factors and to fulfill several objectives (such as frequency allocation and depth control).

In this paper, a technique is proposed for optimizing a circular complementary split ring resonator (CC-SRR) and square complementary split ring resonator (SC-SRR) linked to microstrip transmission lines. The optimization method is developed to precisely assign the operating frequency of the device while enhancing the resonator’s quality factor. In addition to being generic, the approach is distinguished by its computational efficiency. The CC-SRR and SC-SRR are tuned to resonate at 15 GHz to detect changes in the sample’s permittivity, permeability, dielectric, and magnetic loss tangents. The application case study is the prime motivation for choosing 15 GHz as the operating frequency. In the design of microwave sensors, the relative permittivity of the test sample is crucial. For instance, microwave sensors have been built to operate between frequencies of 1 GHz and 6 GHz for measuring samples with high relative permittivity values (*ε_r_* = 20 to 82) [27,28,29]. Microwave sensors are commonly built for frequencies between 7 GHz and 14 GHz for the characterization of samples with medium relative permittivity values (*ε_r_* = 4 to 20) [30,31] and between 14 GHz and 30 GHz for the measurement of low permittivity materials (*ε_r_* = 1 to 4) [32,33]. These examples demonstrate that, depending on the anticipated material qualities of the samples being tested, the required resonant frequency of the sensor may vary significantly. To reliably evaluate oils with a relative permittivity between 2.20 and 3.08, a resonator with a high degree of sensitivity is required. Despite the fact that the challenge of fabrication tolerance is amplified by this high sensitivity. We calibrated our sensor after fabrication to prevent fabrication tolerance from affecting the relative permittivity measurement of oils in order to solve this problem. Both sensors are manufactured using a standard photolithographic procedure and measured using a Rohde & Schwarz ZNB20 vector network analyzer. To establish the fabrication tolerance of the CC-SRR and SC-SRR, the simulated and measured transmission coefficients of the unloaded sensors are compared. According to the comparison, the SC-SRR has a low fabrication sensitivity and outstanding accuracy, making it suitable for the high-resolution characterization of dielectric substrates and oil samples. A polylactide acid (PLA) container is placed over the SC-SRR in the ground plane to confine the oil under test (OUT) and to enable its interaction with the electromagnetic (EM) field emitted from the SC-SRR. Furthermore, upon calibration, a closed-form analytical expression is derived which allows for direct extraction of the OUT’s relative permittivity by measuring the sensor’s real-time transmission response due to interaction with the OUT. 

The main contribution of this paper is a rapid design optimization technique based on surrogate modeling of geometric parameters that outperforms the parameter sweep method, the latter being commonly used in practice to achieve parameter tuning. In fact, identifying an optimal design using parameter sweeping is impossible given a few parameters and various objectives (for example, frequency allocation of the resonance and managing its depth). In contrast, the method provided in this paper can control both objectives, handle several geometry parameters at the same time, and is provably convergent. Furthermore, it has demonstrated the ability to produce sensors with high sensitivity and less measurement error comparable to state-of-the-art circuits.

## 2. Design and Optimization

**Figure 1:**
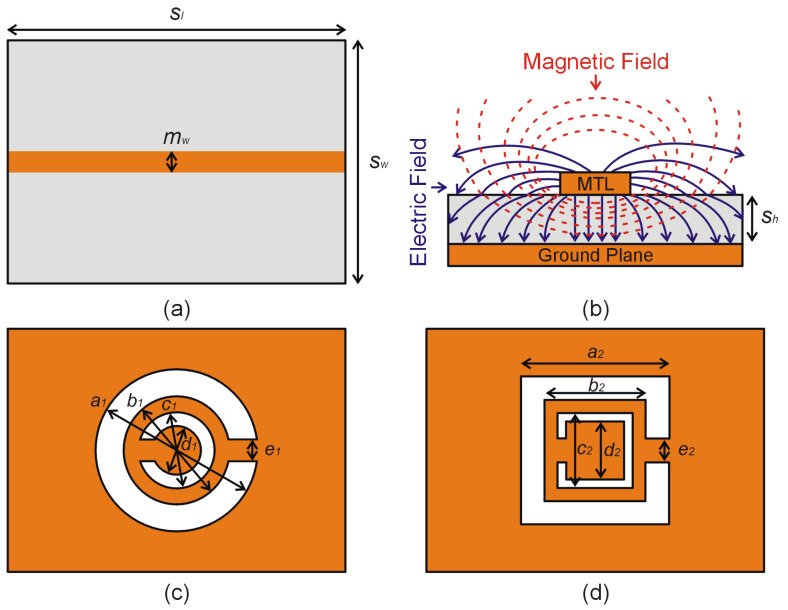
(**a**) Top view of the sensor based on the RO4003C substrate and microstrip transmission line (MTL), (**b**) Electromagnetic fields created by the MTL, (**c**) Geometry of the optimized CC-SRR, and (**d**) Geometry of the optimized SC-SRR.

Two microwave sensors are designed using a circular complementary split ring resonator (CC-SRR) and a square complementary split ring resonator (SC-SRR) connected to a microstrip transmission line (MTL). As illustrated in Figure 1, each sensor is implemented on a RO4003C substrate with a relative permittivity *ε_r_* = 3.38 ± 0.05, length *s_l_* = 30 mm, width *s_w_* = 25 mm, and height *s_h_* = 0.813 mm. The MTL is printed on the top layer of the RO4003C substrate, whereas the CC-SRR and SC-SRR are etched into the bottom layer of each sensor. The following equations are used to optimize the size of the MTL in order to create a 50-ohm sensor impedance that matches the impedance of the vector network analyzer [34].

(1) $$\varepsilon_{re}=\frac{\varepsilon_{r}+1}{2}+\frac{\varepsilon_{r}-1}{2}{\left(1+12\frac{s_{h}}{m_{w}}\right)}^{-0.5}$$

(2) $$Z_{c}=\frac{\eta }{\sqrt{\varepsilon_{re}}}{\left\{\frac{m_{w}}{s_{h}}+1.393+0.677\ln\left(\frac{m_{w}}{s_{h}}+1.4444\right)\right\}}^{-1}$$

where *ε_re_* = 2.67 is the effective dielectric constant of the MTL, *η* = 120π Ω is the impedance of the wave in free space, and the optimized width of the MTL is *m_w_* = 1.88 mm. Figure 1b depicts the electromagnetic field generated by the MTL as a result of waveguide port excitation. Five independent variables explain the geometry of CC-SRR, as illustrated in Figure 1c: *a*_1_ (outer diameter of the outer circle), *b*_1_ (inner diameter of the outer circle), *c*_1_ (outer diameter of the inner circle), *d*_1_ (inner diameter of the inner circle), and *e*_1_ (size of the splits in each circle). As shown in Figure 1d, the geometry of the SC-SRR is described by five parameters: *a*_2_ (outer length of the outer square), *b*_2_ (inner length of the outer square), *c*_2_ (inner length of the inner square), *d*_2_ (outer length of the inner square), and *e*_2_ (size of the splits in each square).

**Figure 2:**
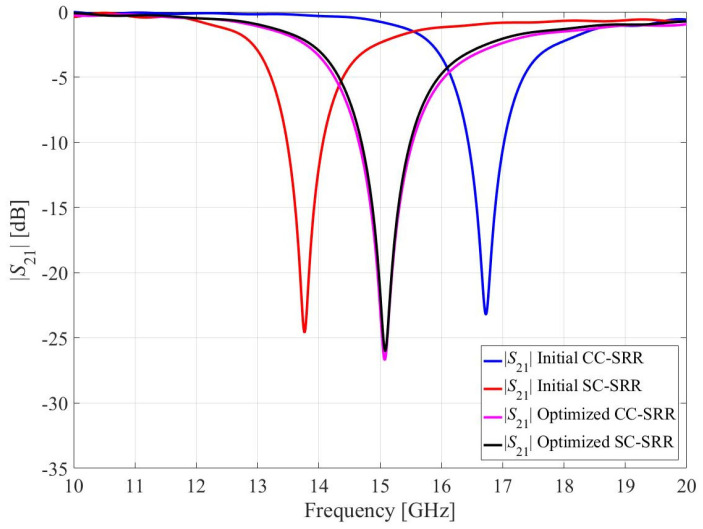
Simulated transmission coefficients for the initial and optimized CC-SRR and SC-SRR.

The initial (prior to optimization) geometric values for the CC-SRR and SC-SRR are *a*_1_ = *a*_2_ = 2 mm, *b*_1_ = *b*_2_ = 1.6 mm, *c*_1_ = *c*_2_ = 1.2 mm, *d*_1_ = *d*_1_ = 0.8 mm, and *e*_1_ = *e*_2_ = 0.2 mm. In CST Microwave Studio, each sensor is simulated using the hexahedral finite integration technique (FIT) solver, and the simulated transmission coefficients (*S*_21_) are shown in Figure 2. According to the simulation results, the CC-SRR has a resonance frequency of 16.72 GHz and a notch depth of −23.19 dB, whereas the SC-SRR has a resonance frequency of 13.77 GHz and a notch depth of −24.58 dB.

**Figure 3:**
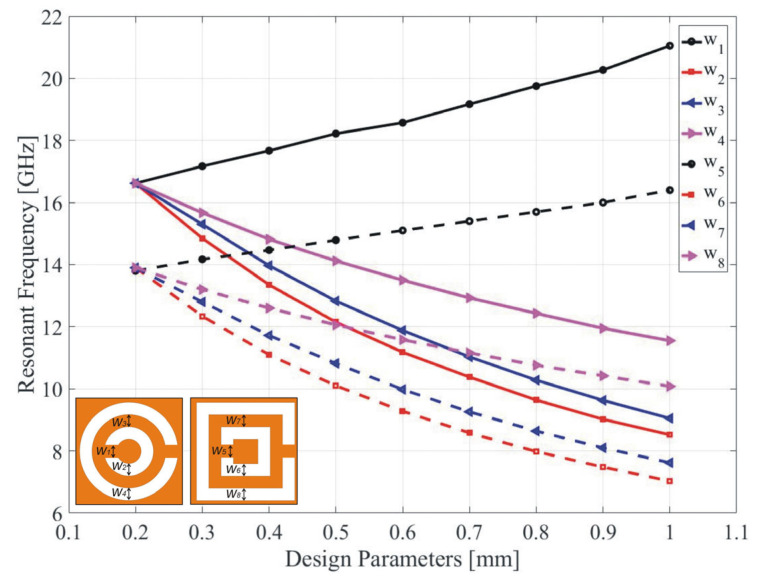
Variation in resonant frequency due to changes in the design parameters of CC-SRR (*w*_1_, *w*_2_, *w*_3_, and *w*_4_) and SC-SRR (*w*_5_, *w*_6_, *w*_7_, and *w*_8_).

**Figure 4:**
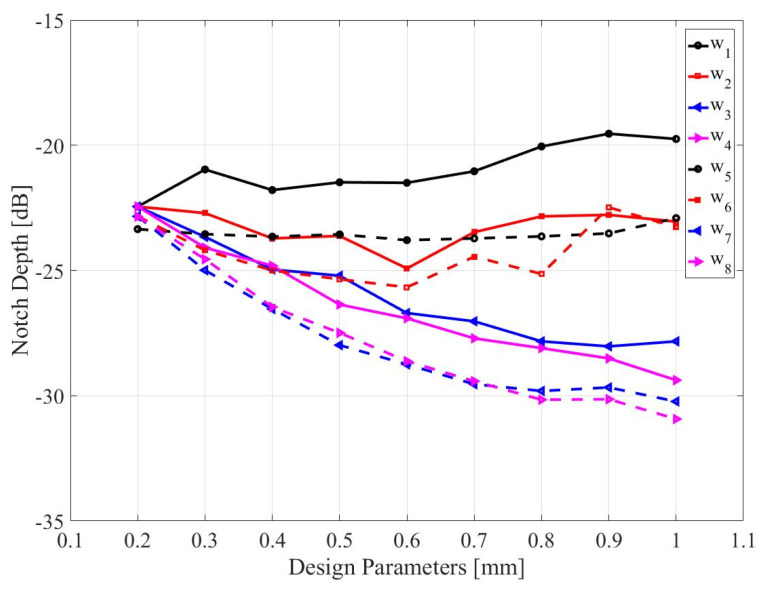
Variation in notch depth due to changes in the design parameters of CC-SRR (*w*_1_, *w*_2_, *w*_3_, and *w*_4_) and SC-SRR (*w*_5_, *w*_6_, *w*_7_, and *w*_8_).

For parametric studies, one parameter is changed at one time while keeping other parameters at their reference values. For this purpose, the CC-SRR is described by variables *w*_1_, *w*_2_, w_3_, and w_4_, whereas the SC-SRR is specified by *w*_5_, *w*_6_, *w*_7_, and *w*_8_. Where *w*_1_ = *e*_1_ = 0.2 mm is the width of the splits for CC-SRR, *w*_2_ = *c*_1_ − *d*_1_/2 = 0.2 mm is the width of the inner ring for CC-SRR, w_3_ = *b*_1_ − *c*_1_/2 = 0.2 mm is the width of copper metal between the inner ring and outer ring for CC-SRR, w_4_ = *a*_1_ − *b*_1_/2 = 0.2 mm is the width of the outer ring for CC-SRR, *w*_5_ = *e*_2_ = 0.2 mm is the width of the splits for SC-SRR, *w*_6_ = *c*_2_ − *d*_2_/2 = 0.2 mm is the width of the inner ring for SC-SRR, *w*_7_ = *b*_2_ − *c*_2_/2 = 0.2 mm is the width of copper metal between the inner ring and outer ring for SC-SRR, and *w*_8_ = *a*_2_ − *b*_2_/2 = 0.2 mm is the width of the outer ring for CC-SRR. The resonant frequency and the notch depth of the CC-SRR and SC-SRR sensors as a result of design parameter variations are shown in Figure 3 and Figure 4, respectively. In these figures, as mentioned earlier, one parameter of each structure is changed at one time while keeping other parameters at their reference values. For example, when the parameter *w*_1_ is changed from 0.2 mm to 1 mm with the step of 0.1 mm, other parameters are kept at their reference values, *w*_2_ = *w*_3_ = *w*_4_ = 0.2 mm.

It can be observed that due to a relatively large number of variables and their joint effects on both the frequency location and the depth of the notch, identifying the optimum solution with respect to the assumed requirements (here, the precise allocation of the notch at the target frequency and maximization of the depth at the same time) is virtually impossible. Further, repetitive sweeps entail considerable computational expenditures due to the massive EM simulations necessary for each tested combination of the parameters. Parameter interdependence, the need for controlling more than one objective at a time, makes simultaneous parameter adjustment imperative, which can only be achieved by means of rigorous numerical optimization techniques.

For the purpose of optimization, the geometric parameters are aggregated into a vector *x* = [*a*_n_ *b*_n_ *c*_n_ *d*_n_ *e_n_*]*^T^*, where *n* = 1 for the CC-SRR and *n* = 2 for the SC-SRR. We are concerned with the resonance frequency of the first notch, denoted as *f*_0_. We also denote as *L*_0_ the level of transmission |*S*_21_| at *f*_0_.

The design optimization task is formulated as:

(3) $$x^{*}=\arg\underset{x}{\min}U\left(x,f_{t}\right)$$

where x^*^ is the optimum design to be identified and *f_t_* is the target notch frequency.

The objective function is defined as:

(4) $$U\left(x,f_{t}\right)=L_{0}\left(x\right)+\beta {\left(f_{t}-f_{0}\left(x\right)\right)}^{2}$$

Its first term is the primary objective (increasing the depth of the resonance), whereas the second term is a penalty function used to enforce and allocate the resonant frequency near the target *f_t_*. Here, we set *f_t_* = 15 GHz. The optimization process is carried out using a trust-region gradient-based algorithm with numerical derivatives [35], which is accelerated using the rank-one Broyden update [36] to reduce the computational cost of the process. Owing to this arrangement, the parameter tuning process is low cost and corresponds to less than thirty EM analyses of the circuit under design. More importantly, the particular formulation problem and the associated definition of the objective function (4) allow for the simultaneous adjustment of all relevant geometry parameters and control over the operating frequency of the structure and the notch depth. Neither of these would be possible using the traditional method, e.g., based on experience-driven parametric studies. Further, the process is fully automated, whereas its computational efficiency cannot be matched by hands-on methods.

The optimization process of CC-SRR leads to *a*_1_ = 2.467 mm, *b*_1_ = 1.726 mm, *c*_1_ = 1.191 mm, *d*_1_ = 0.810 mm, and *e*_1_ = 0.359 mm. The obtained notch depth is –26.67 dB, with the resonant frequency being 15.08 GHz. The SC-SRR optimization results in *a*_2_ = 2.073 mm, *b*_2_ = 1.412 mm, *c*_2_ = 1.052 mm, *d*_2_ = 0.814 mm, and *e*_2_ = 0.336 mm. The notch depth observed is –26 dB and the resonant frequency is 15.08 GHz. Although the optimized resonators have different geometric shapes but the same transmission coefficients, as shown in Figure 2, they can be referred to as equivalent resonators. The optimized resonators have unequal widths in both rings as shown in Figure 1, which distinguishes them from conventional resonators in the literature and affects their sensitivity, which will be discussed in the following section.

## 3. Sensitivity Analysis

The optimized sensors undergo sensitivity analysis by perturbing the SUT’s volume and electromagnetic properties. According to the perturbation technique, the change in resonance frequency (∆*f_r_*) of the sensor is proportional to the perturbed volume (*dυ*), the change in permeability (∆*µ*), and the permittivity (∆*ε*) of the SUT. The following equation expresses the fractional change in the resonant frequency of the sensor [37]:

(5) $$\frac{\Delta f_{r}}{f_{0}}≃\frac{-\int_{V_{0}}(\Delta \varepsilon {\left|{\bar{E}}_{0}\right|}^{2}+\Delta \mu {\left|{\bar{H}}_{0}\right|}^{2})d\upsilon }{\int_{V_{0}}(\varepsilon {\left|{\bar{E}}_{0}\right|}^{2}+\mu {\left|{\bar{H}}_{0}\right|}^{2})d\upsilon }$$

**Figure 5:**
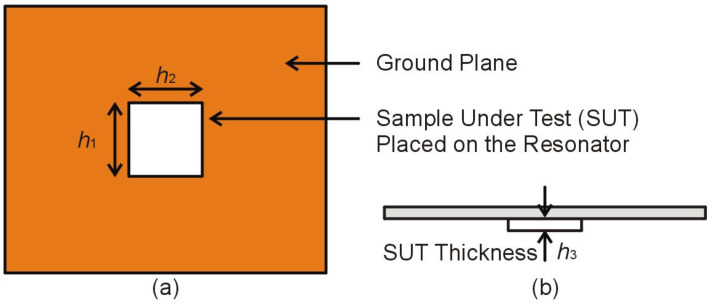
The sensor’s ground plane contains the sample under test (SUT) which interacts with the electromagnetic fields created by the microstrip transmission line and radiated by the resonator, (**a**) Top View (**b**) Front View.

**Figure 6:**
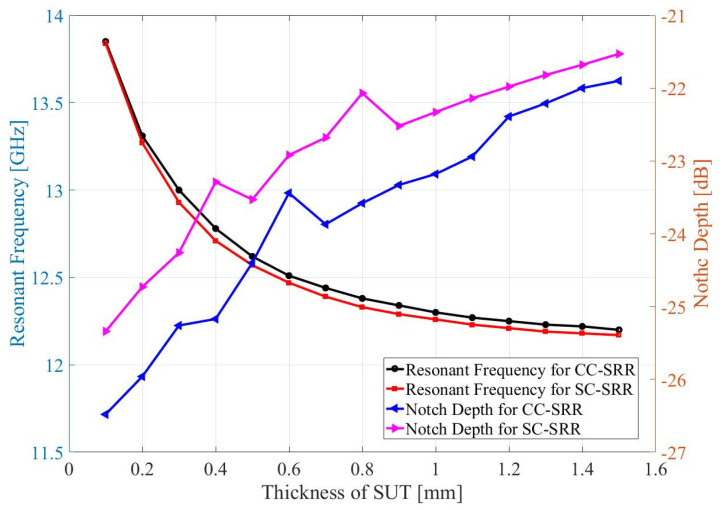
Effect of the SUT’s thickness on CC-SRR and SC-SRR sensors’ resonance frequency and notch depth.

This equation indicates that any increase in the volume, permeability, or permittivity of the SUT will decrease the sensor’s resonant frequency. For volume perturbation, a sample under test (SUT) is placed on the CC-SRR and SC-SRR in each sensor’s ground plane, as illustrated in Figure 5. Because the CC-SRR and SC-SRR are etched out of the ground plane’s copper layer (17.5 μm), there is an air gap between the substrate and the SUT in the shape of resonators, which is taken into account in modeling. A RO4003C substrate with relative permittivity *ε_r_* = 3.38 ± 0.05 is utilized as the SUT (*h*_1_ = 5 mm and *h*_2_ = 5 mm) and the volume of the SUT is changed by altering the thickness (*h*_3_ = 0.1 mm to 1.5 mm) of the RO4003C substrate. Figure 6 shows that the change in resonance frequency is inversely related to the SUT’s thickness up to 1 mm; beyond that, the influence of the SUT’s thickness begins to diminish, and for thickness larger than 1.5 mm, the resonance frequency is saturated. Table 1 summarizes the effect of the SUT’s thickness on the resonance frequency, bandwidth, and loaded Q factor of the CC-SRR and SC-SRR sensors. Increasing the thickness of the SUT reduces the resonant frequency and bandwidth while increasing the sensor’s Q factor. The SC-SRR sensor’s frequency change is larger than that of the CC-SRR sensor, showing that the SC-SRR is more sensitive to changes in the SUT’s thickness.

**Figure 7:**
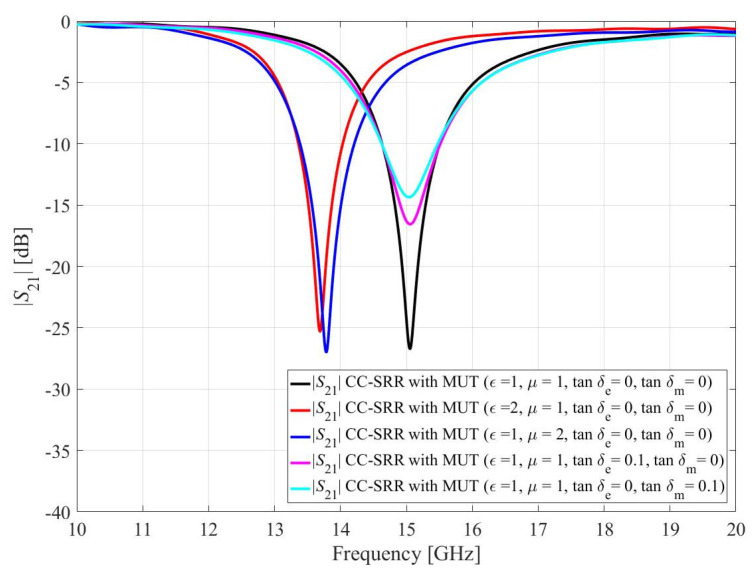
Simulated transmission coefficients (*S*_21_) of the CC-SRR due to interaction with the sample under test (SUT) by perturbing one of SUT’s electromagnetic parameters.

**Figure 8:**
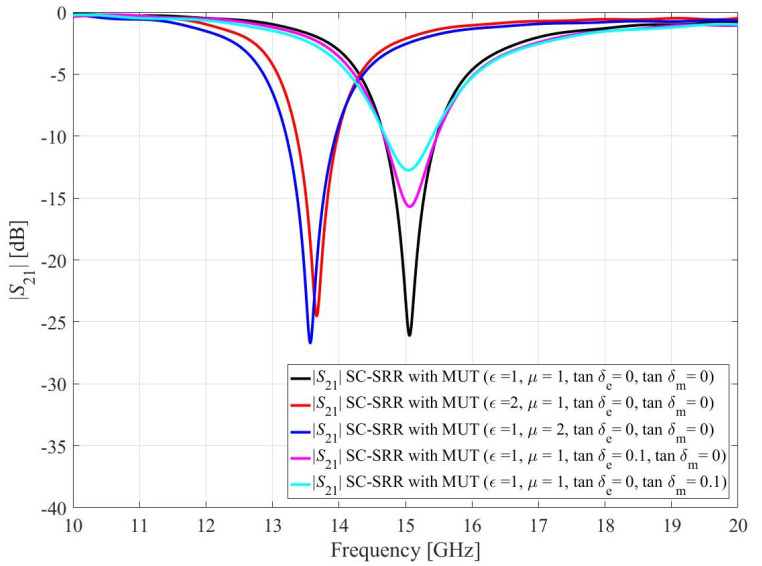
Simulated transmission coefficients (*S*_21_) of the SC-SRR due to interaction with the sample under test (SUT) by perturbing one of SUT’s electromagnetic parameters.

For electromagnetic properties perturbation, the volume of the SUT is kept constant (*h*_1_ = 5 mm, *h*_2_ = 5 mm, and *h*_3_ = 1 mm) and the permittivity (*ε_r_*), permeability (*μ_r_*), dielectric loss tangent (tan*δ_e_*), and magnetic loss tangent (tan*δ_m_*) of the SUT is varied as tabulated in Table 2. According to Table 2, the *ε_r_* and *μ_r_* of the SUT affect the resonance frequency of the sensors while the tan*δ_e_* and tan*δ_m_* of the SUT influence the notch depth of the sensors. Figure 7 and Figure 8 show the effect of the SUT’s electromagnetic properties on the transmission coefficients of the CC-SRR and SC-SRR sensors. Only numerical simulations can reveal the individual effect of electromagnetic characteristics, as shown in Figure 7 and Figure 8. The effect of multiple combinations of EM properties can be seen in measurements where dielectric materials are primarily determined by their relative permittivity; the magnetic permeability is typically one and loss tangents are near zero. The resonator’s high resonance frequency and narrow bandwidth are critical to the performance of a microwave sensor. By optimizing geometrical parameters, the transmission response of both CC-SRR and SC-SRR resonators is made equivalent (same resonance frequency and bandwidth). The size of the resonator is the next factor that influences performance; a smaller size results in a stronger electromagnetic field that interacts with the SUT. Because the optimized SC-SRR is smaller in size than the CC-SRR, it outperforms its circular equivalent in performance.

## 4. Fabrication and Measurement

**Figure 9:**
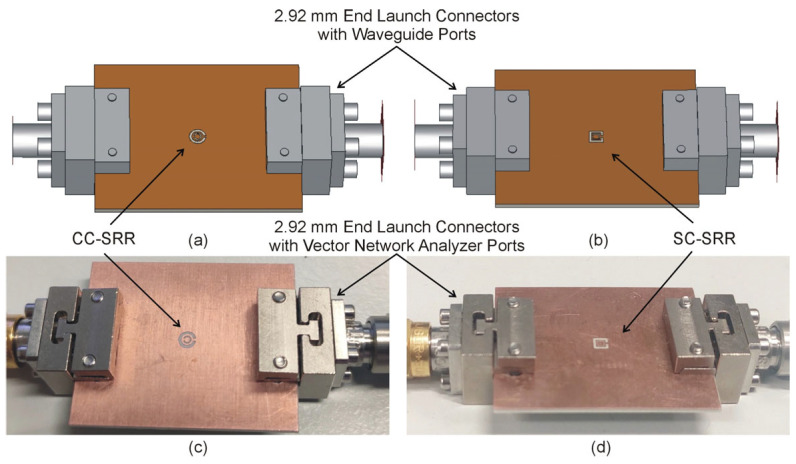
(**a**) Simulated model of optimized CC-SRR sensor with end launch connectors, (**b**) Simulated model of optimized SC-SRR sensor with end launch connectors, (**c**) Fabricated CC-SRR sensor with end launch connectors, and (**d**) Fabricated SC-SRR sensor with end launch connectors.

**Figure 10:**
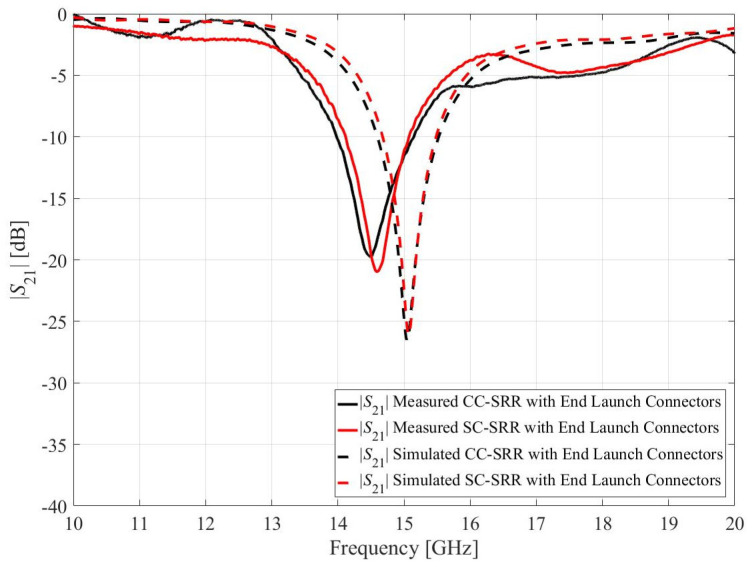
Measured and simulated transmission coefficients (*S*_21_) of the optimized sensors with end launch connectors.

The optimized sensors have been fabricated using the chemical etching process on a double-sided copper-clad laminated (17.5 μm) RO4003C substrate, cf. Figure 9. The dimensions of the substrate, microstrip transmission line, and the optimized CC-SRR and SC-SRR are the same as mentioned in the optimization section. To measure the transmission response (*S*_21_), the fabricated sensors are connected to the vector network analyzer (Rohde & Schwarz ZNB20) using 2.92 mm end launch connectors. As illustrated in Figure 9, the simulation model is implemented using 2.92 mm end launch connectors to determine the influence of end launch connectors on the *S*_21_ of the optimized sensors. The simulated and measured *S*_21_ of the optimized sensors with end launch connectors is shown in Figure 10. The CC-SRR and SC-SRR sensors with end launch connectors have the simulated resonant frequencies of 15.05 GHz with a notch depth of −26.59 dB and 15.07 GHz with a notch depth of −26.16 dB, respectively. For the CC-SRR sensor, the difference between the simulated resonant frequencies with and without end launch connectors is 0.03 GHz, whereas, for the SC-SRR sensor, it is 0.01 GHz. The effect of end launch connectors on resonant frequencies is negligible, although the simulation takes five times longer than with sensors without end launch connectors. The measured resonant frequencies of the CC-SRR and SC-SRR sensors are 14.51 GHz with a notch depth of −19.83 dB and 14.62 GHz with a notch depth of −21.4 dB, respectively. For the CC-SRR sensor, the difference between the simulated and measured resonant frequencies is 3.65%, while for the SC-SRR sensor, it is 3.03%. The better fabrication tolerance of the SC-SRR is due to the structural shape of the resonator. In general, circular shapes are more susceptible to fabrication than square shapes, as demonstrated by our findings. To ensure the consistency of the measured data, each sensor is manufactured twice using the same procedure, and the results are similar. The measured result indicates that the SC-SRR has a good fabrication tolerance than the CC-SRR; therefore, it is calibrated for practical applications, as described in the next section.

## 5. Calibration Procedure and Results

**Figure 11:**
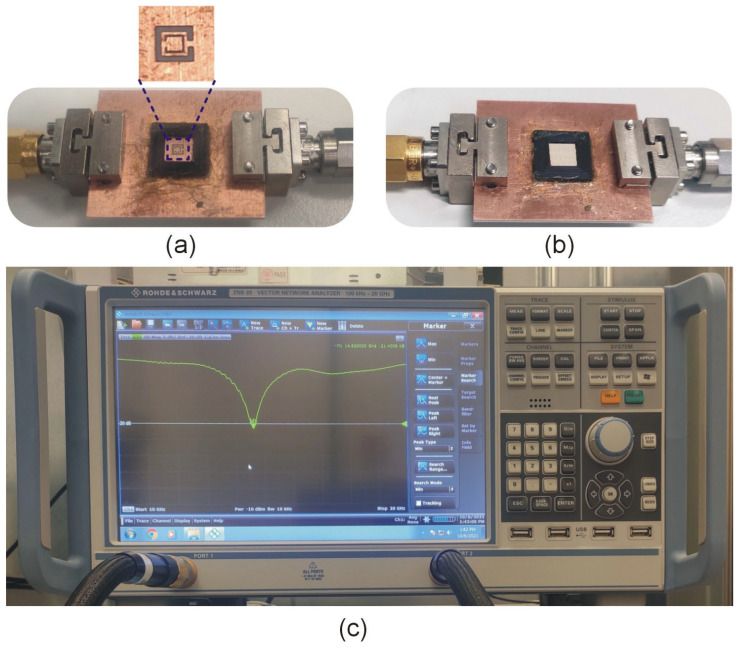
Photographs of the SC-SRR sensor’s fabricated prototype: (**a**) bottom view with PLA container and the optimized CSRR, (**b**) sensor with MUT, and (**c**) Vector network analyzer (VNA) while measuring the unloaded sensor.

A three-dimensional dielectric container (internal size = 5 mm × 5 mm × 1 mm) is incorporated into the design for the positioning of the sample under test (SUT), which enables precision and accuracy for the measurement operations, as illustrated in Figure 11. A polylactide acid (PLA) container is manufactured using a 3D printer owing to the PLA properties of excellent surface quality, decent strength, and low sensitivity to temperature variations. The PLA container is attached to the SC-SRR sensor in the ground plane using cyanoacrylate adhesive, as shown in Figure 11a. To calibrate the sensor, five SUTs with known relative permittivity values, namely TLY-5 (*ε_r_* = 2.2), AD250C (*ε_r_* = 2.5), RO4003C (*ε_r_* = 3.38), RF-35 (*ε_r_* = 3.5), and FR4 (*ε_r_* = 4.3), and fixed dimensions of 5 mm × 5 mm × 1 mm, are placed inside the PLA container, as shown in Figure 11b. The values of the permittivity used for calibration are obtained from the datasheets provided by the manufacturer of these SUT. According to the materials’ datasheet, the uncertainty in permittivity values is +/−0.05. A 1-mm-thick sample is used to eliminate the ambient effect because the impact of the SUT’s thickness begins to diminish after 1 mm (cf. Figure 6). The transmission responses of the sensor loaded with the aforementioned SUTs are measured ten times for each material to minimize stochastic errors. Table 3 shows the obtained results, i.e., the mean and standard deviation of the loaded sensor resonant frequencies. A variety of variables can influence the variation between calibration runs for the same dielectric material. The first is the air gap between the sample and the resonator, the second is the sample condition, and the third is the surrounding environment, such as air humidity or temperature, of the measuring setup. Table 3 illustrates a significant standard deviation in the measured resonance frequency among all ten runs. The frequency shift appears to saturate with larger dielectric constants. The resonant frequency is nearly identical at larger relative dielectric constants (particularly for RF35 and FR4). This indicates that the sensor has a low dynamic range despite its high sensitivity. Instead of a dynamic range, a resonator with extremely high sensitivity is required to evaluate oils with relative permittivity values ranging from 2.22 to 3.08. The data in Table 3 are employed to establish an inverse regression model that permits the direct identification of the unknown permittivity of the SUT, based on the measured resonant frequency *f*_0_.

The model is assumed to have the following analytical form [38]:

(6) $$\varepsilon_{r}=F(f_{0},a)=a_{0}+a_{1}f_{0}+a_{2}f_{0}^{2}$$

Here, *ε_r_* stands for the relative permittivity of the SUT. This particular analytical form is chosen based on the initial analysis of the dependence between the observed resonant frequency and dielectric permittivity of the material. The objective is to employ a simple form parameterized using a small number of coefficients (here, three). On the one hand, this allows for accounting for the weak nonlinearity of the aforementioned relationship. On the other hand, it allows us to maintain the uniqueness of the solution to the regression task, and to smoothen out fluctuations due to measurement errors. The model is parameterized using ***a*** = [*a*_0_ *a*_1_ *a*_2_]*^T^*. This vector is identified by solving the regression tasks *ε_r.j_* = *F*(*f*_0.*j*_,***a***), *j* = 1, …, 5, with *ε_r.j_* and *f*_0.*j*_ being the dielectric permittivity and measured sensor resonant frequency as shown in Table 3. The least-square solution to the above regression problems is equivalent to minimizing *E*(***a***), defined as:

(7) $$E(a)=\left\|{\left[\varepsilon_{r.1}\;\ldots \;\varepsilon_{r.5}\right]}^{T}-{\left[F(f_{0.1},a)\;\ldots \;F(f_{0.5},a)\right]}^{T}\right\|$$

The minimum can be found analytically as:

(8) $$a={\left[A^{T}A\right]}^{-1}A^{T}{\left[\varepsilon_{r.1}\;\ldots \;\varepsilon_{r.5}\right]}^{T}$$

where the *k*th row of the matrix ***A*** is [1 *f*_0.*k*_ *f*_0.*k*_^2^], *k* = 1, …, 5.

Upon substituting the data in Table 3, we find ***a*** = [58.697 −7.207 0.223]*^T^*, i.e., the regression model takes the form of

(9) $$F(f_{0},a)=58.697-7.207f_{0}+0.223f_{0}^{2}$$

**Figure 12:**
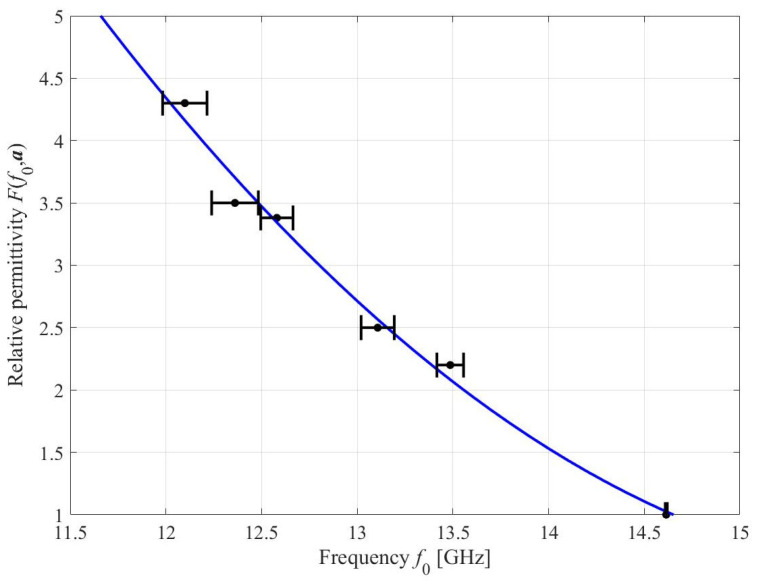
Calibration model of the proposed sensor. Circles correspond to the MUT measurement data in Table 3. Horizontal bars represent standard deviations of the measurement data.

Figure 12 shows the plot of the calibration model along with the error bars corresponding to the standard deviations of the measurement data (cf. Table 3). Based on these, it can be estimated that the sensor measurement error, when using the calibration model, is less than five percent. Five oils with known dielectric characteristics are used to validate the inverse regression model; the extracted oil permittivity values are remarkably similar to those reported in the literature.

## 6. Application Case Study: Oil Measurement

**Figure 13:**
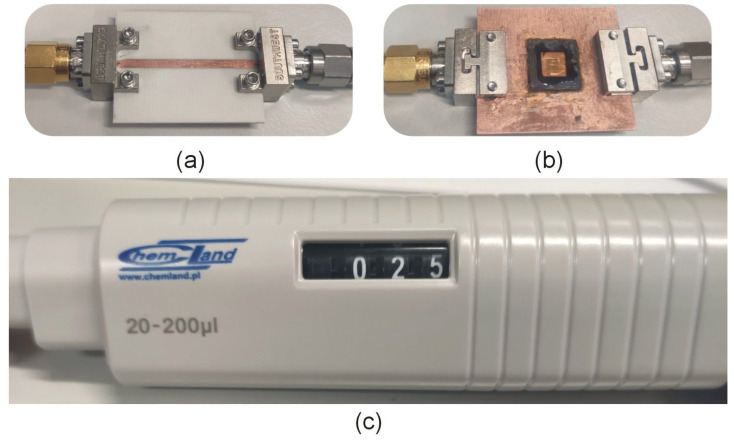
Measurement setup: (**a**) top view of the sensor, (**b**) bottom view with PLA container and oil under test, and (**c**) single-channel adjustable volume pipette used for volume control of the oils.

**Figure 14:**
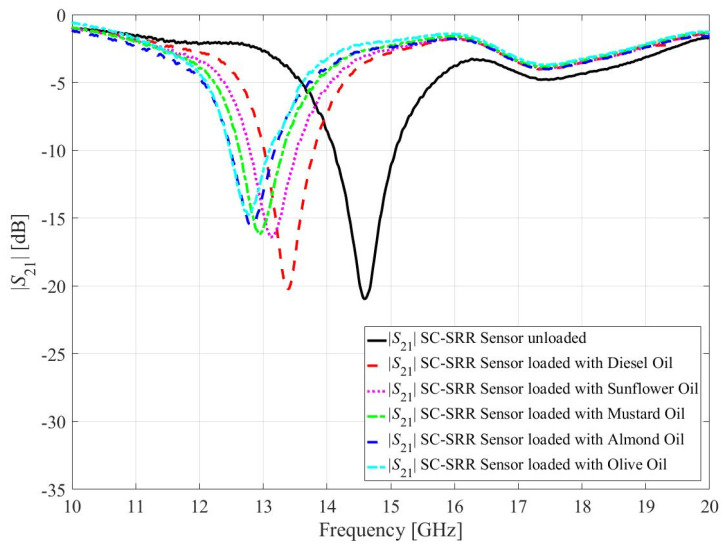
Calibration model of the proposed sensor. Circles correspond to the MUT measurement data in Table 3. Horizontal bars represent the standard deviations of the measurement data.

The calibration model is used to evaluate the relative permittivity of commonly available oils such as diesel oil (*ε_r_* = 2.2) [39], sunflower oil (*ε_r_* = 2.44) [40], mustard oil (*ε_r_* = 2.70) [41], almond oil (*ε_r_* = 3.03) [42], and olive oil (*ε_r_* = 3.08) [42]. For this purpose, 25 μL of each oil was poured into the PLA container as shown in Figure 13. A single-channel adjustable volume pipette is used to control the volume of the oil samples, as shown in Figure 12c. In order to avoid contamination by the previous oil sample, the PLA container is cleaned with alcohol each time, and the alcohol is left to evaporate so that the resonant frequency of the unloaded sensor is not compromised. The sensor’s transmission response due to interaction with the oils was measured and plotted as shown in Figure 14. The resonant frequencies of the optimized sensor due to interaction with diesel engine oil, sunflower oil, mustard oil, almond oil, and olive oil are 13.39 GHz, 13.17 GHz, 12.94 GHz, 12.84 GHz, and 12.76 GHz, respectively. The corresponding relative permittivity of these oils obtained from the calibration model is 2.2, 2.48, 2.80, 2.97, and 3.06, which are close to the values available in the literature.

To compare the performance of the manufactured sensor to that of other sensors, the most important criterion is relative sensitivity. The relative sensitivity of the optimized sensor can be calculated using the following relation [43]:

(10) $$S_{\varepsilon_{r}}=\frac{f_{u}-f_{l}}{f_{u}\left(\varepsilon_{r}-1\right)}\times 100$$

where *f_u_* is the sensor’s resonant frequency without SUT and *f_l_* is the resonant frequency while interacting with the SUT of relative permittivity *ε_r_*.

The optimized sensor provides a minimum frequency shift of 1.23 GHz and a maximum sensitivity of 7.01 percent due to interaction with the diesel oil. According to (10), the sensitivity is directly proportional to the resonant frequency of the sensor. While the resonant frequency is inversely proportional to the permittivity of the substrate. As a result, if we use a different substrate with high relative permittivity, such as FR4 (*ε_r_* = 4.3), the resonant frequency of the same sensor will decrease, resulting in a decrease in sensor sensitivity. Table 4 compares the relative sensitivity of the sensor proposed in this work and the state-of-the-art sensors reported in the literature. It can be observed that the proposed device clearly outperforms the benchmark in terms of relative sensitivity.

## 7. Conclusions

This article presented an optimization and inverse-model-based calibration technique for circular complementary split ring resonators (CC-SRR) and square complementary split ring resonators (SC-SRR) linked to microstrip transmission lines. The CC-SRR and SC-SRR are described using five independent parameters and these variables are meticulously tuned to allocate the resonant frequency of the sensors at 15 GHz. Each sensor is subject to sensitivity analysis by altering the permittivity, permeability, dielectric, and magnetic loss tangents of the sample under test after obtaining the equivalent transmission response using the optimization technique. This approach cannot be generalized to lossy properties or non-unitary magnetic permeability values because practical materials are categorized according to their dielectric constant. The effect of 2.92 mm end launch connectors on the transmission responses of unloaded optimized sensors is numerically calculated, revealing that they have a maximum effect of 0.03 GHz on the resonance frequencies, despite the fact that the simulation takes five times longer than with sensors without end launch connectors. Fabrication tolerance is a crucial component that causes the simulation and measurement to disagree. The fabrication tolerance of the CC-SRR and SC-SRR is investigated by comparing the unloaded sensors’ simulated and measured transmission coefficients. The discrepancy between the simulated and measured resonance frequencies is 3.65 percent for the CC-SRR sensor and 3.03 percent for the SC-SRR sensor. The fabricated sensor based on SC-SRR is calibrated through measurements of dielectric samples of known permittivity and the inverse regression model. For the positioning of the sample under test, a three-dimensional dielectric container is incorporated into the design, providing precision and reliability for the measurement procedures. Application case studies carried out for several oil samples demonstrate competitive sensitivity of 7.01 percent for the optimized sensor, outperforming the state-of-the-art sensors reported in the literature.

## Figures and Tables

**Table 1:** The influence of the SUT’s thickness on the resonant frequency, bandwidth, and Loaded Q factor of the CC-SRR and SC-SRR sensors.

Rogers RO4003C (5 × 5 mm)	Circular Complementary Split Ring Resonator (CC-SRR)			Square Complementary Split Ring Resonator (SC-SRR)		
Thickness of Sample Under Test (mm)	Resonance Frequency (GHz)	Bandwidth (GHz)	Loaded Q Factor	Resonance Frequency (GHz)	Bandwidth (GHz)	Loaded Q Factor
0.1	13.85	2.95	4.69	13.84	1.91	7.24
0.2	13.31	1.89	7.04	13.27	1.69	7.85
0.3	13.00	1.73	7.51	12.93	1.56	8.28
0.4	12.78	1.66	7.69	12.71	1.47	8.64
0.5	12.62	1.57	8.03	12.57	1.43	8.79
0.6	12.51	1.53	8.13	12.47	1.40	8.90
0.7	12.44	1.51	8.23	12.39	1.38	8.97
0.8	12.38	1.48	8.36	12.33	1.36	9.06
0.9	12.34	1.47	8.39	12.29	1.34	9.17
1.0	12.30	1.46	8.42	12.26	1.33	9.21
1.1	12.27	1.45	8.46	12.23	1.33	9.19
1.2	12.25	1.44	8.50	12.21	1.32	9.25
1.3	12.23	1.44	8.49	12.19	1.32	9.23
1.4	12.22	1.44	8.48	12.18	1.32	9.22
1.5	12.20	1.44	8.47	12.17	1.32	9.21

**Table 2:** The influence of the electromagnetic properties of the SUT on the resonance frequency and notch depth of the CC-SRR and SC-SRR sensors.

Sample Under Test (SUT)				Transmission Coefficient (*S*_21_) for CC-SRR		Transmission Coefficient (*S*_21_) for SC-SRR	
*ε_r_*	*µ_r_*	*tanδ_e_*	*tanδ_m_*	{GHz}	{dB}	{GHz}	{dB}
1	1	0	0	15.05	−26.73	15.06	−26.12
2	1	0	0	13.69	−25.31	13.67	−24.53
1	2	0	0	13.78	−27	13.57	−26.72
1	1	0.1	0	15.05	−16.55	15.06	−15.69
1	1	0	0.1	15.05	−14.35	15.06	−12.75

**Table 3:** Measurement results for the calibration materials.

Materials for Calibration	Relative Permittivity (*ε_r_*)	Measured Resonant Frequency	
		Mean {GHz}	Standard Dev. {GHz}
TLY-5	2.2	13.49	0.069
AD250C	2.5	13.11	0.086
RO4003C	3.38	12.36	0.084
RF-35	3.5	12.10	0.122
FR4	4.3	12.09	0.116

**Table 4:** Comparative analysis of contemporary sensors based on optimization, calibration method, and relative sensitivity.

Ref.	Resonator Architecture	Sample Under Test (SUT)	Optimization of Geometric Parameters	Calibration Method	Resonant Frequency {GHz}	Relative Sensitivity (%)
[43]	OCSRR	DI Water, butanol, ethanol, and methanol	No	Curve Fitting	0.33	0.504
[44]	SRR	Micro fluids	Yes	N.A	1.72	0.78
[45]	SIR	Water and methanol	No	Curve Fitting	1.91	0.84
[46]	SSRR	Rogers 5880, Rogers 4350, and FR-4	No	Polynomial Fittings	2.22	1.51
[47]	SRR	Foam and FR4 polyethylene	No	N.A	1.8	3.04
[48]	CSRR	Oil samples	No	N.A	2.5	3.58
[49]	CSRR	Roger substrates	No	N.A	5.39	5.54
[50]	SRR	Lubricating oil and iron powder	No	Curve Fitting	7.69	3.45
[51]	CMSRR	Vegetable oils	No	Curve Fitting	7.2	5.21
[52]	CSSSR	Teflon and glass	No	Curve Fitting	15.12	6.7
[53]	Dielectric Rod Resonator	Single crystal sapphire, polycrystalline ceramics, and cordierite	No	N.A	60	3.27
[54]	Dielectric Resonator	Distilled water, bacteriostatic water, saline, and methanol	No	N.A	2.3	1.6
[55]	Dielectric Resonator	Ethanol-water solution	No	N.A	2.48	0.04
This Work	Square CSRR	Dielectric substrates and oils	Yes	Inverse Regression Model	14.62	7.01

## Data Availability

Not applicable.
